# Clinical characterization of a novel *ATP1A2* p.Gly615Glu mutation in nine family members with familial hemiplegic migraine

**DOI:** 10.1093/braincomms/fcae447

**Published:** 2024-12-10

**Authors:** Marina Romozzi, Serena Spartano, Federica Francesca L’Erario, Luigi Francesco Iannone, Vincenzo Trigila, Annalisa Gentile, Pasquale Sanginario, Paolo Calabresi, Francesco Danilo Tiziano, Catello Vollono

**Affiliations:** Dipartimento Universitario di Neuroscienze, Università Cattolica del Sacro Cuore, 00168 Roma, Italy; Neurologia, Dipartimento di neuroscienze, Organi di Senso e Torace, Fondazione Policlinico Universitario Agostino Gemelli IRCCS, 00168 Rome, Italy; Section of Genomic Medicine, Department of Life Sciences and Public Health, Catholic University of Sacred Heart and Complex Unit of Medical Genetics, Fondazione Policlinico Universitario Agostino Gemelli IRCCS, 00168 Rome, Italy; Section of Genomic Medicine, Department of Life Sciences and Public Health, Catholic University of Sacred Heart and Complex Unit of Medical Genetics, Fondazione Policlinico Universitario Agostino Gemelli IRCCS, 00168 Rome, Italy; Section of Clinical Pharmacology and Oncology, Department of Health Sciences, University of Florence, 50134 Florence, Italy; Dipartimento Universitario di Neuroscienze, Università Cattolica del Sacro Cuore, 00168 Roma, Italy; Dipartimento Universitario di Neuroscienze, Università Cattolica del Sacro Cuore, 00168 Roma, Italy; Dipartimento Universitario di Neuroscienze, Università Cattolica del Sacro Cuore, 00168 Roma, Italy; Neurologia, Dipartimento di neuroscienze, Organi di Senso e Torace, Fondazione Policlinico Universitario Agostino Gemelli IRCCS, 00168 Rome, Italy; Dipartimento Universitario di Neuroscienze, Università Cattolica del Sacro Cuore, 00168 Roma, Italy; Neurologia, Dipartimento di neuroscienze, Organi di Senso e Torace, Fondazione Policlinico Universitario Agostino Gemelli IRCCS, 00168 Rome, Italy; Section of Genomic Medicine, Department of Life Sciences and Public Health, Catholic University of Sacred Heart and Complex Unit of Medical Genetics, Fondazione Policlinico Universitario Agostino Gemelli IRCCS, 00168 Rome, Italy; Dipartimento Universitario di Neuroscienze, Università Cattolica del Sacro Cuore, 00168 Roma, Italy; Neurologia, Dipartimento di neuroscienze, Organi di Senso e Torace, Fondazione Policlinico Universitario Agostino Gemelli IRCCS, 00168 Rome, Italy

**Keywords:** hemiplegic migraine, *ATP1A2*, cortical spreading depression, episodic ataxia

## Abstract

Familial hemiplegic migraine type 2 results from pathogenic variants in the *ATP1A2* gene, which encodes for a catalytic subunit of sodium/potassium ATPase. This extremely rare autosomal dominant disorder manifests with a spectrum of symptoms, most commonly pure hemiplegic phenotype, epilepsy, and/or intellectual disability. In this study, we detail the clinical features and genetic analysis of nine patients from a large family spanning four generations, with all carrying a previously unreported likely pathogenic variant, p.Gly615Glu, in *ATP1A2*, compatible with a diagnosis of familial hemiplegic migraine type 2, fully penetrant with variable expressivity. This newly identified likely pathogenic variant primarily presented with psychiatric disturbances and a non-hemiplegic phenotype. Only one patient presented hemiplegic attacks, while seven were diagnosed with migraine with aura, including visual, sensory, and speech/language aura, and one with migraine without aura. The identification of the genes responsible for the more common forms of migraine, both with and without aura, remains a significant challenge in migraine genetics and is critical for advancing personalized medicine.

## Teaser

Romozzi *et al*. describe a family of nine members with familial hemiplegic migraine Type 2 caused by a novel likely pathogenic variant (p.Gly615Glu) in the *ATP1A2* gene. The variant demonstrated full penetrance with variable expressivity, predominantly presenting with psychiatric disturbances and migraine with aura instead of hemiplegic attacks.

## Graphical Abstract

**Graphical abstract:**
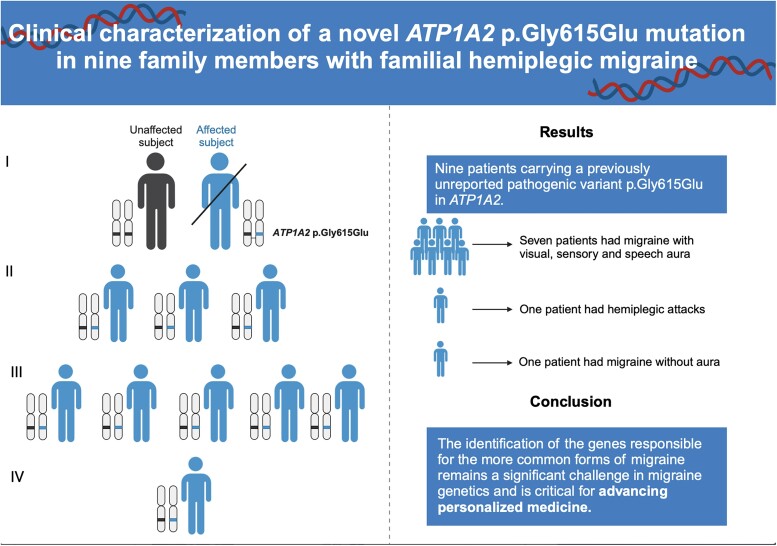

## Introduction

Hemiplegic migraine (HM) is a rare form of migraine consisting of both reversible motor weakness and visual, sensory, or speech symptoms, and it can occur as a sporadic or familial disorder.^1^ Familial HM (FHM) is a very rare autosomal dominant disorder caused by pathogenic variants (PVs)/likely PVs (LPVs) in at least four distinct genes that encode proteins involved in ion transport [*CACNA1A* (OMIM: 601011), *ATP1A2* (OMIM: 182340), *SCN1A* (OMIM: 182389) and *PRRT2* (OMIM: 614386)].^1,2^ The known PVs account for a small percentage of cases of FHM, being other genes not yet identified.^2^ PVs related to HM increase neuronal excitability, leading to a higher propensity to cortical spreading depression/depolarization (CSD), which is the pathophysiological mechanism underlying the aura.^3^

The clinical presentation of FHM attacks is highly variable. Severe attacks can be accompanied by fever, seizures, coma, signs of encephalopathy, cerebral oedema, cerebellar involvement, and long-lasting motor impairment.^4,5^ Furthermore, permanent symptoms, such as cerebellar ataxia, can develop interictally in patients with HM and often depend on the specific gene involved.^1,2^

PVs in *ATP1A2*, which encodes for a catalytic subunit of sodium/potassium ATPase, are responsible for FHM Type 2 (FHM2, OMIM: 602481).^6^ The clinical spectrum ranges from migraine without aura to migraine with typical aura and pure hemiplegic phenotype, variably associated with epilepsy and/or intellectual disability.^7-9^

Herein, we describe the clinical characteristics and genetic analysis of nine patients belonging to a large kindred having an unreported *ATP1A2* PV, with a prevalent non-hemiplegic phenotype associated with psychiatric disturbances.

## Materials and methods

The study was carried out in compliance with the Helsinki Declaration and with the guidelines of the Ethical Committee of our Institution. Informed consent was obtained from all the participants in the study. Written informed consent was obtained from all patients about the storage and use of their DNA samples for both diagnostic and clinical research studies concerning their disease.

The proband belongs to a four-generation family including 14 individuals who were visited in the outpatient headache centre of Fondazione Policlinico Universitario Agostino Gemelli in Rome.

Two headache specialists (M.R. and C.V.) interviewed and collected clinical data of each family member, confirming the diagnosis of migraine and the type according to the International Classification of Headache Disorders, third edition (ICHD-3).^1^ The following clinical and demographic data were collected in patients diagnosed with migraine: age, sex, comorbidities, migraine characteristics (including the presence and characterization of aura, disease duration, and onset of migraine), and monthly headache days. Additionally, patients completed the Headache Impact Test (HIT-6) and the Migraine Impact and Disability Assessment Scale questionnaires.

**Figure 1:**
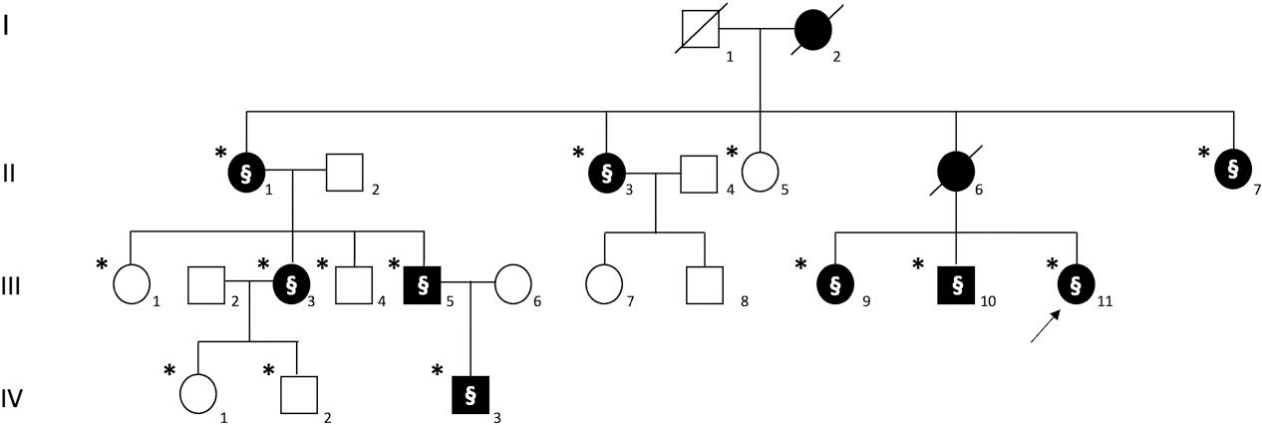
**FHM Type 2 pedigree chart of the family.** Full symbols represent affected subjects. The asterisk symbol denotes available DNA sample for testing; the section symbol denotes the presence of the ATP1A2:c.1844G > A LPV. I, first generation; II, second generation; III, third generation; and IV, fourth generation.

For mutational analysis, genomic DNA was extracted by standard salting-out procedure, from blood samples of the 14 individuals (marked by the asterisks in Fig. 1), 9 affected and 5 asymptomatic.

We performed Next Generation Sequencing (NGS) analysis of a panel of the five genes indicated in Table 1, in our index case only (III,11 in Fig. 1). For library preparation, we used the AmpliSeq Technology (Thermofisher) with a custom panel, and the Personal Genome Machine (Thermofisher) for sequencing analysis, according to the manufacturer’s instructions. The mean depth of the run was 1825×, 99.93% of the target sequence covered at least at 20×, and 99.90% at 100×. Variants were annotated by the IonReporter online tool (IonTorrent, Thermofisher). Variants having a Minimum Allele Frequency > 0.1% were filtered out, as well as, synonymous and intronic variants (unless potentially modifying exon splicing).

**Table 1:** List of genes included in the NGS panel

Gene	Reference transcript	No. of exons	No. of amplicons	Size of sequence/gene (bp)	Gene size (bp)	Open reading frame size (bp)
*SCN1A*	NM_006920.6	29	48	9066	143 380	5997
*CACNA1A*	NM_023035.3	47	73	14 475	300 038	7539
*SCN2A*	NM_001040142.2	27	48	13 161	152 891	6018
*ATP1A3*	NM_152296.5	23	36	9415	276 497	3042
*ATP1A2*	NM_000702.4	23	29	6269	27 833	3063

NGS panel, transcript reference, total number of exons, number of amplicons per gene in the panel, no. of amplicon for each gene, no. of sequenced bp, length of the entire gene, and length of coding sequence.

The candidate *ATP1A2* variant found, was confirmed by Sanger sequencing of PCR products (ATP1A2-ex14-F:AGACATCTCGCTATCTAGCTTTCTC; ATP1A2-ex14-R:TCACAATAGTGTCAGAGGTAAGGC), as previously described.^10^

### Statistical analysis

Continuous variables were reported as mean ± standard deviation. All data analyses were performed using SPSS software version 26.0 (IBM Corp. SPSS Statistics, Armonk, NY, USA).

## Results

The proband (Fig. 1, III,11) was a 22-year-old female with a history of migraine characterized by visual, sensory, and speech auras, followed by headache starting from the age of 17. One episode of a severe migraine attack required hospitalization in our Institution. Brain MRI, magnetic resonance angiography, and electroencephalography performed outside of migraine attacks (EEG) were unremarkable.

The family history was positive for headache. The proband’s mother, who died from melanoma (II,6, Fig. 1), experienced migraine attacks with visual, sensory, and speech/language auras from the youth.

The proband’s maternal aunt (63-year-old; Fig. 1, II,1) has had recurrent attacks of visual, sensory, and speech/language auras with a weekly frequency starting from the age of 10. Additionally, from the age of 50, she experienced three focal seizure episodes with loss of consciousness. Following her last seizure attack at the age of 62, she was prescribed lamotrigine, with no further seizure episodes and a marked reduction in the number of aura events. She was also diagnosed with panic disorder, without receiving any specific treatment. Her two sisters (Fig. 1, II,3 and II,7) also experienced visual and sensory auras with a weekly frequency. One of these (Fig. 1, II,3) was diagnosed with bipolar disorder at the age of 40, and treated with valproic acid, and the other sister with major depressive disorder (Fig. 1, II,7).

The proband’s older brother (Fig. 1, III,10) had experienced prolonged speech and sensory auras since the age of 12, with two attacks requiring hospitalization.

The proband’s eldest sister (Fig. 1, III,9) has had HM since the age of 5, characterized by hemiparesis, sensory deficits, and aphasia lasting <60 min, followed by headache. The motor aura occurred approximately once per month, while migraine without aura or non-motor auras had a frequency of eight times per month.

In the third generation, the proband’s cousin (Fig. 1, III,5) experienced recurrent attacks of migraine with visual and sensory auras weekly, while his 12-year-old son (Fig. 1, IV,3) had migraine without aura at a frequency of once per month since the age of 8. Additionally, subject III,3, a 41-year-old woman, has had frequent attacks of migraine with visual, sensory, and speech/language auras weekly and daily headaches since the age of 14.

**Figure 2:**
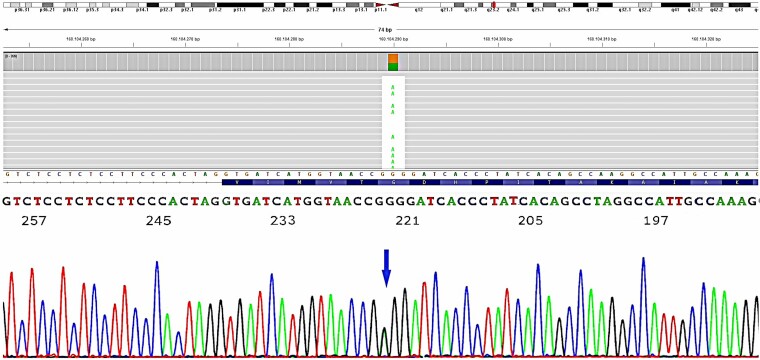
**Sequencing analysis of ATP1A2 of the index case.** (**A**) Integrative Genomics Viewer (https://igv.org/) picture of the LPV identified in the index case by NGS, and (**B**) Sanger sequencing confirmation of the variant (indicated by the arrow).

We identified in the proband the heterozygous *ATP1A2*:c.1844G > A, p.(Gly615Glu) variant, located in exon 14 (Fig. 2). This variant is not reported either in the gnomAD (v4.1, https://gnomad.broadinstitute.org) or ClinVar (https://www.ncbi.nlm.nih.gov/clinvar/) databases. In the same aminoacidic position, another PV [*ATP1A2*: c.1843G > A, p.(Gly615Arg)] was previously identified.^11,12^ In synthesis, the c.1844G > A was classified as LPV (Class IV) since fulfilling the PM2, PM5, PP1, and PP3 criteria, according to the American College of Medical Genetics classification.^13^ The same PV was present in all subjects screened displaying signs compatible with the diagnosis of migraine with and without aura and HM (9/14). Of the nine cases, six were females (66.7%), and three were males (22.3%). The mean age of onset was 12.8 ± 3.1 (range, 8–17), and the mean age at examination was 38.7 ± 17.3 (range, 12–63).

The other five family members tested negative for the LPV and reported no headache or aura, except for one who was diagnosed with tension-type headache. Given that tension-type headaches occur in up to 50% of the general population, this case was considered within the expected range.

Clinical characteristics of the nine cases carrying *ATP1A2* mutation are summarized in Table 2.

**Table 2:** Demographic and clinical characteristics of the nine patients carrying the *ATP1A2* mutation

	Sex	Age	ICHD-3 diagnosis	Age of onset	MHD	Type of aura	Aura monthly frequency	HIT-6	MIDAS	Maximum duration of attacks (days)	Triggers of attacks	Psychiatric comorbidities
**II,1**	F	63	MA	10	8	V, S, L	4	72	50	4	Stress	Panic disorder, depression
**II,3**	F	61	MA	14	7	V, S	10	59	35	2	Stress, menstrual cycle, food	Bipolar disorder
**II,7**	F	51	MA	10	12	V, S	4	54	7	1	Bright lights, sleep deprivation, food	Depression
**III,3**	F	41	MA	14	22	V, S, L	15	62	34	10	Food, bright lights, menstrual cycle	
**III,5**	M	34	MA	13	30	V, S	5	63	33	5	Fasting	Insomnia, depression
**III,9**	F	37	HM	17	8	V, S, L, Mo	8	72	55	7	Stress	Depression, anxiety
**III,10**	M	27	MA	12	15	S, L	<1	40	1	0.5	Stress	Anxiety
**III,11**	F	22	MA	17	5	V, S, L	<1	46	1	1	Stress	
**IV,3**	M	12	MO	8	4					1		

F, female; HIT-6, Headache Impact Test-6; HM, hemiplegic migraine; ICHD-3, International Classification of Headache Disorders, third edition; L, speech/language aura; M, male; Mo, motor; MHD, monthly headache days; MIDAS, migraine disability assessment; MO, migraine without aura; S, sensory aura; V, visual aura.

Attacks of HM fulfilling the criteria of the ICHD-3^1^ were reported by only one of nine subjects (11.1%). Seven subjects carrying the PV had a diagnosis of migraine with typical aura, and one patient only had attacks of migraine without aura. Emotional stress, menstruation, and some specific foods were the most frequent triggering factors.

Only one patient bearing the pathogenic variants (PV) suffered from concomitant epilepsy. Psychiatric disturbances (detailed in Table 2) were present in 6/9 patients carrying the PV (66.7%). The nine patients’ brain MRIs were unremarkable and did not specifically show signs of cerebellar atrophy.

## Discussion

We report a large family with FHM2 and the unreported PV p.Gly615Glu in *ATP1A2*, fully penetrant and with variable expressivity. The prevalent clinical expression was migraine with non-motor aura and comorbid psychiatric disturbances.

Besides FHM2,^6^ the clinical spectrum of *ATP1A2* ranges from migraine with or without aura, intellectual disability, and seizures in ∼15% of the cases, including benign familial infantile convulsions and generalized epilepsy with febrile seizures.^7-9,14^ Two *ATP1A2* germline variants were also identified in patients with alternating hemiplegia of childhood.^15^

The *ATP1A2* gene encodes the α2 subunit of Na^+^/K^+^-ATPase, which is a membrane-integral protein belonging to the P-type ATPase family. In the adult brain, *ATP1A2* is predominantly expressed in astrocytes.^7^ Moreover, the α2 Na^+^/K^+^-ATPase is also colocalized alongside the excitatory amino acid transporters in the astrocytic plasma membrane.^16^ Different FHM phenotypes exhibit varying degrees of Na^+^/K^+^-ATPase loss of function, impairing the reuptake of potassium and glutamate in the synaptic cleft, leading to brain hyperexcitability and increased susceptibility to CSD, which is the pathophysiological substrate of aura and epileptic discharges.^7^

Furthermore, in our family, psychiatric disturbances were frequent, reported by six out of eight adult patients, even in the absence of a hemiplegic phenotype. Castro *et al*. reported a novel *APT1A2* mutation in a Portuguese family. The proband, along with the mother, exhibited mood disorders and borderline personality disorder, all within the context of a very severe hemiplegic phenotype accompanied by encephalopathy.^17^

Similarly, a preclinical study utilizing knock-in mice, heterozygous for the G301R mutation, demonstrated that the α2+/G301R mice exhibited various behavioural phenotypes relevant to FHM with comorbid psychiatric disturbances.^18^ The authors suggest that these phenotypes are most likely linked to an imbalance in the glutamate system, probably involving the *N*-methyl-D-aspartate receptor, resulting in reduced glutamate clearance by astrocytes involving the α2 Na^+^/K^+^-ATPase.^18^

None of the patients in the family experienced permanent motor, sensory, language, or visual symptoms. Only one patient was also diagnosed with epilepsy, characterized by a very low frequency of seizures. This condition went untreated for years until lamotrigine was prescribed, after which no further seizures occurred. Additionally, there was a significant reduction in the number of aura events, likely attributed to the anti-glutamatergic effects of lamotrigine, which may indirectly influence CSD.^19^

## Conclusion

Our data suggest that the prevalent clinical manifestations of the *ATP1A2* c.1844G > A PV is migraine with typical aura associated with psychiatric disorders. Identifying the genes that cause the more common forms of migraine, including migraine with typical aura and migraine without aura, remains a significant challenge in migraine genetics and is critical for advancing personalized medicine.

## Data Availability

The data collected are available from the corresponding author upon reasonable request.
